# Occupational exposure to total and hexavalent chromium in welders: an integrated environmental and biological monitoring study

**DOI:** 10.3389/fpubh.2026.1830673

**Published:** 2026-05-19

**Authors:** Andrea Spinazzè, Veruscka Leso, Salvatore Della Notte, Carolina Zellino, Andrea Cattaneo, Sandro Recchia, Carlo Dossi, Elena Grignani, Domenico Maria Cavallo, Ivo Iavicoli

**Affiliations:** 1 Department of Science and High Technology, University of Insubria, Como, Italy; 2 Department of Public Health, University of Naples “Federico II”, Naples, Italy; 3 Department of Theoretical and Applied Sciences, University of Insubria, Varese, Italy; 4 Environmental Research Center, Istituti Clinici Scientifici Maugeri IRCCS, Pavia, Italy; 5 Section of Occupational Medicine, Department of Healthcare Surveillance and Bioethics, Catholic University of Sacred Heart, Rome, Italy; 6 Fondazione Policlinico Universitario A. Gemelli, IRCCS, Rome, Italy

**Keywords:** biological monitoring, exhaled breath condensate, occupational exposure assessment, respirable particulate matter, risk assessment, stainless steel, urinary chromium, welding fumes

## Abstract

**Introduction:**

Chromium (Cr), and particularly its hexavalent form is classified as a Group 1 human carcinogen and represents a major occupational hazard, particularly in stainless-steel welding. This exploratory study integrated environmental and biological monitoring to characterize welders' exposure and assessed how analytical methods influence exposure estimates.

**Methods:**

Twenty-two welders from two Northern Italian companies and 10 administrative controls were enrolled (May–June 2025) and monitored during full 8 h shifts. Personal respirable particulate samples were analyzed for total Cr (CrTOT) and Cr(VI) using NIOSH 7,600 and a speciation-preserving modified ISO 17075 protocol. Biological monitoring included pre-/post-shift urinary Cr (U-Cr, creatinine-adjusted) and post-shift exhaled breath condensate Cr (EBC-Cr).

**Results:**

All airborne CrTOT and Cr(VI) concentrations were below their respective Occupational Exposure Limit Values (500 and 5 μg/m^3^, respectively), though welders consistently showed higher exposure than controls. MMA welding produced the highest CrTOT and Cr(VI) levels. NIOSH 7600 yielded higher Cr(VI) values than the modified ISO method, a pattern consistent with a possible positive bias related to species interconversion during extraction. U-Cr was significantly elevated in welders at both sampling times [geometric mean (GM): 0.34, 0.36, and 0.05 μg/g creatinine in pre-, post-shift exposed workers and controls, respectively], whereas EBC-Cr did not differ between groups (GM: 0.08 μg/L in all samples). Biomarker levels showed minimal variation across demographic and occupational subgroups. Correlations between airborne Cr and biological indicators were weak, yet U-Cr values in welders exceeded population reference ranges, indicating a clear occupational contribution despite low absolute exposure.

**Discussion:**

Integrated monitoring confirmed Cr absorption in welders despite regulatory compliance. Within the limits of this study, U-Cr appeared to be the most informative biomarker, whereas EBC-Cr showed limited sensitivity. Although exposures remained below biological limit values, welders exhibited an upward shift in U-Cr relative to background populations, supporting continued exposure minimization-especially for high-emission processes such as MMA-consistent with As Low As Reasonably Achievable principles.

## Introduction

Chromium (Cr) is a heavy metal extensively used in numerous industrial applications. Despite its technological relevance, several Cr compounds are associated with adverse health effects in humans, including allergic dermatitis, asthma, pneumonitis, pharyngitis, and an increased risk of lung cancer among exposed workers (1–5). Occupational exposure to hexavalent chromium [Cr(VI)] falls within the scope of legislation governing carcinogenic and mutagenic agents in the workplace (6, 7).

Exposure to Cr(VI) may occur in a variety of occupational settings, such as welding, Cr(VI) electroplating, and other surface treatment processes, including the application and removal of Cr-containing paints (2). Welding and flame cutting of stainless steel generate Cr-oxide particles containing both trivalent [Cr(III)] and Cr(VI) species. The composition of welding fumes largely depends on the technique applied, with Manual Metal Arc (MMA) welding generally producing higher Cr(VI) emissions than Metal Inert Gas (MIG) or Tungsten Inert Gas (TIG) processes (4). In addition, physicochemical properties of Cr(VI)-containing particles, such as solubility and particle size distribution, influence exposure likelihood, uptake pathways, and Cr toxicokinetic (1, 7).

Environmental monitoring of occupational Cr(VI) exposure traditionally relies on active personal sampling of airborne particulate matter, followed by analytical determination of Cr(VI) concentrations and comparison with appropriate Occupational Exposure Limits (OELs). However, several analytical challenges complicate accurate exposure assessment, particularly with regard to reliable detection, quantification, characterization, and maintenance of Cr(III)/Cr(VI) speciation. These challenges can be broadly categorized into three key aspects: (i) method sensitivity in relation to low sample mass, (ii) the redox stability of Cr(VI) and Cr(III) under extraction conditions, and (iii) appropriate sampling and storage strategies for collected particulate matter (8). The most widely adopted standardized methods for determining Cr(VI) in airborne particulate matter include ISO 16740:2005, NIOSH 7600–7605, and OSHA ID-215. These procedures typically involve sampling on PVC filters, hot alkaline extraction, and colorimetric detection using 1,5-diphenylcarbazide. Nevertheless, such methods may lack robustness, as elevated temperature and alkaline conditions can promote oxidation of Cr(III) to Cr(VI), potentially leading to overestimation of exposure levels. In light of these limitations, analytical approaches capable of monitoring and correcting Cr(III)/Cr(VI) interconversion during extraction are warranted. Speciated Isotope Dilution Mass Spectrometry (SIDMS) provides a suitable solution by employing isotopically enriched tracers (e.g., ^53^Cr and ^50^Cr) to track and correct redox transformations occurring during sample preparation and analysis. Within this context, a novel analytical procedure based on liquid chromatography coupled with inductively coupled plasma mass spectrometry (LC-ICP-MS) was developed to enhance sensitivity compared with conventional colorimetric techniques and to enable SIDMS-based correction of species interconversion. A dedicated chromatographic separation strategy was implemented to manage elevated Cr(III) concentrations, and a new extraction protocol, inspired by ISO 17075, was optimized under near-neutral pH conditions at room temperature to preserve chromium speciation. This approach was previously applied to particulate matter samples from the leather industry, confirming the limitations of official protocols (9).

With respect to biomonitoring of occupational Cr(VI) exposure, total Cr in urine (U-Cr) represents the most commonly used biomarker in occupational medicine practice. However, although U-Cr is considered a sensitive indicator of internal Cr dose, it lacks sufficient specificity to discriminate between exposure to different Cr(III) and Cr(VI) compounds, which exhibit distinct toxicokinetic and toxicodynamic profiles (2). This limitation reduces its utility for refined risk assessment and highlights the need to identify more specific biomarkers of Cr(VI) exposure across different biological matrices. Among these, Cr measured in exhaled breath condensate (EBC-Cr) represents a promising biomarker, providing information on Cr levels at the pulmonary level, the primary target organ of inhaled Cr(VI) (10). EBC sampling is non-invasive, repeatable, and well suited for field studies in occupational settings, allowing assessment of metals deposited in the airway lining fluid. Early investigations demonstrated that both total Cr (11, 12) and Cr(VI) (11, 13) could be detected in the EBC of Cr plating workers and were closely correlated (11). However, the proportion of Cr(VI) relative to total Cr was shown to decrease over time since the last exposure, suggesting partial reduction of inhaled Cr(VI) within the pulmonary lining fluid (13). In stainless-steel TIG welders, total Cr in EBC was proposed as a reliable exposure marker (14), and subsequent studies on welders have further supported the applicability of EBC for monitoring exposure to welding fumes, showing associations between airborne metal concentrations and EBC metal levels, as well as short-term variations related to work shifts (10, 11). These findings indicate that EBC-Cr may reflect recent pulmonary deposition and early biological interactions at the respiratory interface. At the same time, they underline the importance of standardized collection procedures, control of potential contamination, and careful interpretation of speciation data, particularly in complex exposure scenarios such as welding, where mixed metal aerosols are generated.

In this context, the primary objective of the present study was to achieve an integrated characterization of occupational Cr exposure by combining advanced environmental and biological monitoring approaches. Environmental and biological data were obtained from the same workers and within the same exposure scenarios, enabling direct correlation between external exposure and internal dose indicators. This combined design allowed evaluation of exposure–dose relationships under real working conditions and supported assessment of the effectiveness of personal protective equipment within the occupational risk management framework. This integrated framework allows a more coherent interpretation of exposure–dose relationships and provides insights to inform updated occupational risk assessment and management strategies.

## Materials and methods

### Company selection and participant recruitment

Two mechanical engineering companies, both located in Northern Italy (Company A and Company B), participated in the study for a total of 22 welders (Company A, *n* = 11; 10 males and 1 female; Company B, *n* = 11 males), with a mean age of 40 ± 10 years. A control group composed of 10 administrative workers (Company A, *n* = 5, of which three males, two females; Company B, *n* = 5 males), with a mean age of 41 ± 13 years, was enrolled to determine baseline exposure levels. The study protocol was reviewed and approved by the Local Ethics Committee Campania 3 (n. 47/2025). All subjects were recruited on a voluntary basis from May to June 2025 and gave written informed consent.

The welding techniques, electrodes and alloys used by each worker are reported in Table 1. The TIG technique was employed by all the welders from Company A. The main metal constituents of the alloy used, the 304 L, a widely employed “18–8” chromium-nickel austenitic stainless steel, were Cr, Fe, Ni, Mn, and Si. In Company B, different welding techniques were used: TIG, and ESW (ElectroSlag Welding). In this latter Company, various types of alloys were used, with a metal composition mainly based on Cr, Ni, Fe, Mo, Mn and Si. Duration of work shifts was 8 h a day for 5 consecutive days. The working areas of both Companies were equipped with both general and local exhaust ventilation (LEV). All welders at both Companies wore personal protection equipment (PPE) including protective half-helmet, welding gloves and fire/flame resistant clothing.

**Table 1:** Welding techniques and materials by each worker.

Company	ID	Welding technique	Welded material	% Cr in welded material	Welding gas mixture	% Cr content of filler material
A	ID-01	Automatic TIG	n.a.	18	Ar+He	22
A	ID-10	Automatic TIG	stainless steel	23	Ar	23
A	ID-10 R	Automatic TIG	stainless steel	23	Ar	23
A	ID-03	Manual TIG	stainless steel	22	Ar	16
A	ID-06	Manual TIG	stainless steel	25	Ar	25
A	ID-06 R	Manual TIG	stainless steel	25	Ar	25
A	ID-07	Manual TIG	stainless steel	22	Ar	25
A	ID-02	MMA	stainless steel	22	–	22
A	ID-04	MMA	stainless steel	18	–	15
A	ID-09	MMA	stainless steel	22	–	22
A	ID-9 R	MMA	stainless steel	22	–	22
A	ID-15	MMA	stainless steel	22	–	22
B	ID-18	Automatic TIG	stainless steel	25	Ar	25
B	ID-25	Automatic TIG	stainless steel	25	Ar	25
B	ID-20	ESW	carbon steel	0	–	23
B	ID-21	ESW	carbon steel	0	–	23
B	ID-28	ESW	carbon steel	0	–	23
B	ID-29	ESW	carbon steel	0	–	23
B	ID-22	Manual TIG	stainless steel	20	Ar	29
B	ID-24	Manual TIG	stainless steel	29	98% Ar + 2% N	29
B	ID-26	Manual TIG	stainless steel	29	98% Ar + 2% N	29
B	ID-27	Manual TIG	stainless steel	29	98% Ar + 2% N	29
B	ID-30	Manual TIG	stainless steel	20	Ar	29

TIG, tungsten inert gas; ESW, electroslag welding; MMA, manual metal arc; n.a, data not available.

### Information collected

At enrolment, all participants completed a structured questionnaire administered by trained study personnel. The questionnaire was designed to collect information relevant to Cr exposure assessment, including demographic characteristics, lifestyle factors, and detailed occupational history. Recorded variables comprised age, sex, smoking status, and other potential determinants of metal exposure. For occupationally exposed workers, additional data were obtained on job title, duration of employment, years of welding activity, average daily working hours, specific tasks performed, and use of personal protective equipment. For control subjects, occupational history was collected to verify the absence of professional exposure to Cr and welding-related activities. The collected information was used to characterize the study population and to support the descriptive and stratified interpretation of environmental and biological monitoring findings. Questionnaires were administered in a standardized manner to ensure completeness and internal consistency.

### Exposure assessment - environmental monitoring

For the purpose of the study, only inhalation exposure route was considered. Personal air samples of the respirable fraction of airborne particles were collected in the breathing zone of selected workers for the assessment of inhalation exposure. Sampling was performed at a flow rate of 3 L/min with a Plastic cyclone and cassette (SKC Inc. 863 Valley View Road Eighty-Four (PA), USA) fitted with pre-weighed PVC filters (PALL-GLA-5,000 low-ash PVC membranes; 25 mm in diameter; porosity 5 μm). Furthermore, respirable particles were also monitored by fixed-site sampling in different spots of the working area; these sampling lines were placed at the same time and place of personal sampling. Workers involved in welding (using various techniques) materials with a high chromium content were selected because of their expected exposure to chromium compounds and Cr(VI). Each sampling campaign consisted of both personal sampling and fixed-site sampling: pairs of samples collected in parallel (duplicates) through personal monitoring were obtained from the two selected companies. Monitored workers were divided into two “Similar Exposure Groups” (SEGs), according to EN 689 (15). The first (SEG A) and second (SEG B) SEG included workers involved in welding activities in the first and second companies under investigation, respectively. Further, 4 pairs of duplicates from fixed-site environmental sampling were collected contextually. The air samples were first analyzed gravimetrically to determine the concentration of airborne respirable particle fraction. After that, the samples were analyzed for Cr(VI) using both the NIOSH 7600 protocol and the modified ISO 17075 protocol as described in Spinazzè et al. (9). In addition, CrTOT was estimated from the analytical workflow used in this study, enabling a method-consistent comparison across chromium metrics.

Details of the analytical procedure, including reagents, extraction according to the NIOSH 7600 and modified ISO 17075 protocols, oxidation-reduction potential (ORP) measurements, chromatographic separation, ICP-MS detection, quantification of natural Cr(VI) and isotopic ^50^Cr(VI)/^53^Cr(III) spikes, and quality assurance/quality control, have been reported in the article describing the method development and its first application (9). Briefly, the modified ISO 17075 workflow involved a 48-h extraction at room temperature in a pH 8 phosphate buffer under orbital shaking, rather than hot alkaline extraction, to better preserve chromium speciation and improve recovery of insoluble Cr(VI). Before extraction, isotopically enriched ^50^Cr(VI) and ^53^Cr(III) spikes were added to each sample to monitor possible Cr(III)/Cr(VI) interconversion during sample preparation. Within this SIDMS-based approach, recovery of the ^50^Cr(VI) spike was used to assess Cr(VI) losses, whereas oxidation of the ^53^Cr(III) spike indicated artifactual Cr(III)-to-Cr(VI) conversion. Extracts were then analyzed by LC-ICP-MS after chromatographic separation, supporting more reliable Cr(VI) quantification in Cr(III)-rich particulate samples. Results of exposure monitoring were used for risk characterization, performed by testing compliance with the European Occupational Exposure Limit Value (OELV) for Cr(VI). Compliance with OELV was statistically evaluated by comparing it with the upper confidence limit (UCL) of 70% with the 95th percentile of the distribution of at least six measurements. If the UCL is lower than the OELV, it is concluded that the probability of exceeding the OELV is acceptably low, that is a condition of compliance with the limit value.

### Exposure assessment - biological monitoring

Two spot urine samples were obtained from exposed workers: the first prior to the start of the initial shift of the working week and the second at the end of the final shift of the same week (usually Thursday or Friday). To minimize the risk of external contamination, participants were instructed to remove work clothing and wash their hands thoroughly before sample collection. For control subjects, one spot urine sample was collected at any time during the working week. Urine samples were collected in decontaminated containers (Becton, Dickinson and Company, NJ, USA). Following collection, samples were homogenized, aliquoted into pre-labeled tubes, and stored at−20 °C until analysis. Urinary creatinine was determined for each sample, and Cr concentrations were adjusted for creatinine to account for urine dilution. Sample analysis was performed using ICP-MS on a PerkinElmer NexION 2000B instrument (PerkinElmer Inc., Waltham, MA, USA).

EBC was collected using the TurboDECCS system (Medivac, Parma, Italy) according to a previously adopted procedure (11, 12). In exposed workers, two EBC samples were obtained: one prior to the beginning of the first shift of the working week and a second at the end of the last shift of the same week (on Thursday). In the control group, a single EBC sample was collected during the working week. To prevent degradation or redox interconversion between Cr(III) and Cr(VI), EBC samples were immediately treated with an Ethylenediaminetetraacetic Acid (EDTA) solution and stored under refrigerated conditions (without freezing). Immediately after collection, an aliquot of each EBC sample was diluted 1:10 with 0.5 mM EDTA, with the pH adjusted to 8 using a 10% v/v ammonia solution. Because the volume of EBC collected may differ among individuals, the measured Cr concentration can be influenced by sample volume. As no validated volume normalization marker is currently available for EBC, results were expressed as μg/L relative to the collected EBC volume. To ensure accurate calculation, the volume of EBC aliquoted for EDTA complexation was recorded, and the remaining non-complexed portion of the sample was weighed upon arrival at the analytical laboratory. Sample analysis was performed using ICP-MS on a PerkinElmer NexION 2000B instrument (PerkinElmer Inc., Waltham, MA, USA).

### Statistics

Data referring to environmental monitoring are presented as mean, median, minimum and maximum values. The distribution of exposure data was confirmed as log-normal using the Shapiro–Wilk test. The Mann-Whitney test was applied to assess if significant differences were present in the concentrations found in samples collected from exposed and control subjects, and also to evaluate differences between Cr(VI) observed in welders using the TIG welding technique and those using the other techniques. The significance level of *p* = 0.05 level and the SPSS statistical software version 22.0 (SPSS, Chicago, IL, USA) were used for statistical analyses. Results were interpreted against international baseline benchmarks and occupational BLVs (Biological limit values) from bodies like Italian Society of Reference Values (SIVR), German Biological Reference Values (DFG BAR), American Conference of Governmental Industrial Hygienists (ACGIH), French Agency for Food, Environmental and Occupational Health & Safety (ANSES) and Instituto Nacional de Seguridad y Salud en el Trabajo (INSST). Biological monitoring data were collected in a Microsoft Excel file (Microsoft, Redmond, Washington, USA), which included information on the analytical results, and were collected and processed anonymously, being linked only to unique study codes. The dataset was then imported into GraphPad Prism software, version 8.0.2 (GraphPad Software), for statistical analysis. The study population was stratified according to several demographic and lifestyle-related variables, including gender, age, smoking habits, area of residence, and extra-occupational activities potentially influencing Cr exposure. Descriptive statistics were computed for all variables. The distribution of data was assessed using the Shapiro-Wilk. Comparisons between independent groups were performed using Student's t-test or the Mann–Whitney U test, as appropriate based on data distribution. For paired observations Wilcoxon signed-rank test was applied. Differences among multiple groups were assessed using one-way analysis of variance (ANOVA) followed by Tukey's *post-hoc* test, or, in the case of non-normally distributed data, the Kruskal–Wallis test with Dunn's *post-hoc* multiple comparisons. The choice between parametric and non-parametric tests was based on data distribution and sample size considerations. Specifically, parametric tests were applied only when the assumptions of approximate normality and homogeneity of variance were met, as assessed through the Shapiro–Wilk test and visual inspection of distribution plots. When these assumptions were violated, or when subgroup sample sizes were too small to ensure the robustness of parametric estimators, non-parametric alternatives were used to provide more reliable inference. This approach ensured that statistical comparisons were aligned with the empirical characteristics of the dataset and minimized the risk of biased or unstable estimates. In accordance with EN 689:2019, exposure data were first examined graphically on a log-probability plot, obtained by ranking measurements in ascending order and plotting them against their cumulative probabilities, to assess approximate log-normality and the consistency of the SEG. Since a minority of measurements was < LOQ, these values were not removed or replaced with fixed substitutions (e.g., LOQ/2). Instead, the Annex H of EN 689:2019 procedure was followed: the distribution was estimated from results above the LOQ, and the < LOQ observations were retained as censored data for subsequent interpretation and compliance testing. Furthermore, to evaluate the relationships between airborne Cr concentrations and biological monitoring parameters, as well as between different biological matrices and Cr content in welding filler materials, Spearman correlation analyses were performed. A two-sided *p*-value < 0.05 was considered statistically significant.

## Results

### Environmental monitoring

Concerning environmental exposure, Tables 2, 3 present the results of personal environmental monitoring for Cr(VI) and total chromium (CrTOT), comparing exposed welders with non-exposed controls. Exposure measurements were classified into SEGs in accordance with EN 689 (15). SEG A and SEG B include workers from the two companies investigated. Data showed log-normal distribution across SEGs, supporting percentile-based descriptors and parametric estimation of upper confidence limits.

**Table 2:** Results of environmental sampling to determine Cr(VI) and CrTOT exposure values, distinguishing between types of subjects (exposed – ID# and controls – IDC#).

ID	Cr(VI) - ISO (μg/m^3^)	Cr(VI) - NIOSH (μg/m^3^)	CrTOT - ISO (μg/m^3^)	CrTOT - NIOSH (μg/m^3^)
ID-01	0.0001	–	0.038	–
ID-02	1.232	0.112	2.264	0.287
ID-03	0.004	0.011	0.032	0.098
ID-04	0.005	0.009	0.312	1.890
ID-06	0.0001	0.015	0.072	0.110
ID-06 R	0.0001	0.034	0.073	0.310
ID-07	0.005	0.017	0.233	0.097
ID-09	0.0001	0.014	0.031	0.114
ID-10	0.00010	0.022	0.040	0.059
ID-10 R	0.0001	0.038	0.048	0.108
ID-11	0.0001	0.006	0.028	0.058
ID-14	0.00003	0.102	0.043	0.430
ID-15	0.078	0.505	0.678	3.492
ID-18	0.001	0.071	0.118	0.197
ID-20	0.001	0.024	0.016	0.103
ID-21	0.003	0.047	0.021	0.315
ID-22	0.0003	0.066	0.113	0.182
ID-24	0.0004	0.114	0.163	0.262
ID-25	0.008	0.127	0.090	0.315
ID-26	0.0002	0.046	0.024	0.162
ID-27	0.006	0.084	0.079	0.242
ID-28	0.0001	0.044	0.024	0.177
ID-29	0.0001	0.046	0.051	0.222
ID-30	0.0001	0.055	0.510	0.222
ID-9 R	0.047	0.134	0.305	0.293
ID-C1	0.00005	0.025	0.012	0.079
ID-C10	0.00005	0.023	0.013	0.146
ID-C2	0.00005	0.005	0.011	0.068
ID-C3	0.00005	0.034	0.013	0.126
ID-C4	0.00005	0.014	0.011	0.077
ID-C5	0.00005	0.022	0.013	0.092
ID-C6	0.00005	0.009	0.011	0.11
ID-C7	0.00005	0.04	0.011	0.131
ID-C8	0.00005	0.044	0.009	0.107
ID-C9	0.012	0.083	0.011	0.18
Stationary 1	0.0002	0.002	0.028	–
Stationary 2	0.0022	0.090	0.074	0.406
Stationary 3	0.0007	0.041	0.032	0.153
Stationary 4	0.00003	0.011	0.014	0.094

The results highlighted in gray are below the limit of quantification (LOQ) of the analytical method.

**Table 3:** Descriptive statistics of results of environmental sampling to determine Cr(VI) and CrTOT exposure, distinguishing between types of subjects (exposed and controls).

Workers group	Analysis method (μg/m^3^)	*N*	Mean (μg/m^3^)	SD	Min (μg/m^3^)	Median (μg/m^3^)	Max (μg/m^3^)
Control	Cr(VI) - ISO	10	0.0013	0.004	0.00005	0.00005	0.012
	Cr(VI) - NIOSH	10	0.03	0.023	0.005	0.024	0.083
	CrTOT - IOS	10	0.012	0.001	0.009	0.011	0.013
	CrTOT - NIOSH	10	0.112	0.035	0.068	0.109	0.18
Exposed	Cr(VI) - ISO	29	0.048	0.228	0.00003	0.0003	1.232
	Cr(VI) - NIOSH	29	0.067	0.094	0.002	0.045	0.505
	CrTOT - ISO	29	0.192	0.428	0.014	0.051	2.264
	CrTOT - NIOSH	29	0.385	0.708	0.0580	0.197	3.492

The results highlighted in gray are below the limit of quantification (LOQ) of the analytical method. TIG, tungsten inert gas; ESW, electroslag welding; MMA, manual metal arc.

Cr(VI) was quantified using NIOSH 7600 and a modified ISO 17075 method optimized to preserve Cr speciation. Significant inter-method differences emerged (*p* < 0.05), with NIOSH generally yielding higher concentrations. A total of 29 respirable samples were analyzed due to repeated measurements for selected workers. Using the modified ISO 17075 protocol, welders showed significantly higher Cr(VI) levels than controls (*p* < 0.05). Among exposed workers, mean ± SD CrTOT was 0.192 ± 0.428 μg/m^3^ (ISO) and 0.385 ± 0.708 μg/m^3^ (NIOSH), while Cr(VI) averaged 0.048 ± 0.228 μg/m^3^ (ISO) and 0.067 ± 0.094 μg/m^3^ (NIOSH). Controls exhibited substantially lower values, confirming occupational exposure relative to background levels.

Table 4 stratifies exposure by welding technique. MMA welding produced markedly higher CrTOT and Cr(VI) concentrations than TIG, consistent with known emission profiles. For MMA, CrTOT reached 0.7179 ± 0.8943 μg/m^3^ (ISO) and 1.2150 ± 1.4630 μg/m^3^ (NIOSH), with Cr(VI) of 0.2723 ± 0.5374 μg/m^3^ (ISO) and 0.1551 ± 0.2037 μg/m^3^ (NIOSH). TIG welding - both manual and automatic - showed substantially lower levels across both methods.

**Table 4:** Descriptive statistics of results of environmental sampling to determine Cr(VI) and CrTOT exposure, distinguishing exposure values according to the type of welding performed by the exposed subjects.

Welding technique	Analysis method (μg/m^3^)	*N*	Mean (μg/m^3^)	SD	Min (μg/m^3^)	Median (μg/m^3^)	Max (μg/m^3^)
Automatic TIG	Cr(VI) - ISO	5	0.0019	0.0034	0.0001	0.0001	0.0080
	Cr(VI) - NIOSH	4	0.0647	0.0461	0.0222	0.0549	0.1267
	CrTOT - ISO	5	0.0668	0.0355	0.0384	0.0480	0.1178
	CrTOT - NIOSH	4	0.1699	0.1125	0.0591	0.1526	0.3153
Manual TIG	Cr(VI) - ISO	9	0.0017	0.0023	0.0001	0.0003	0.0059
	Cr(VI) - NIOSH	9	0.0490	0.0346	0.0109	0.0465	0.1136
	CrTOT - ISO	9	0.1444	0.1520	0.0244	0.0786	0.5105
	CrTOT - NIOSH	9	0.1872	0.0772	0.0970	0.182	0.3103
MMA	Cr(VI) - ISO	5	0.2723	0.5374	0.0001	0.0469	1.2320
	Cr(VI) - NIOSH	5	0.1551	0.2037	0.0094	0.1123	0.5052
	CrTOT - ISO	5	0.7179	0.8943	0.0311	0.3117	2.2639
	CrTOT - NIOSH	5	1.2150	1.4630	0.1138	0.2926	3.4916
ESW	Cr(VI) - ISO	4	0.0011	0.0015	0.0001	0.0007	0.0032
	Cr(VI) - NIOSH	4	0.0404	0.0109	0.0241	0.0453	0.0470
	CrTOT - ISO	4	0.0278	0.0155	0.0161	0.0222	0.0506
	CrTOT - NIOSH	4	0.2044	0.0888	0.1030	0.1996	0.3154
Not specified	Cr(VI) - ISO	2	0.00007	0.00005	0.00003	0.00007	0.0001
	Cr(VI) - NIOSH	2	0.0538	0.0676	0.0060	0.0538	0.1016
	CrTOT - ISO	2	0.0353	0.0102	0.0281	0.0353	0.0426
	CrTOT - NIOSH	2	0.2436	0.2631	0.0576	0.2436	0.4296
Control (no welding activities performed)	Cr(VI) - ISO	10	0.0012	0.0038	0.0001	0.0001	0.0120
	Cr(VI) - NIOSH	10	0.0299	0.0226	0.0050	0.0240	0.0830
	CrTOT - ISO	10	0.0115	0.0013	0.0090	0.0110	0.0130
	CrTOT -NIOSH	10	0.1116	0.0350	0.0680	0.1085	0.1800

**Figure 1:**
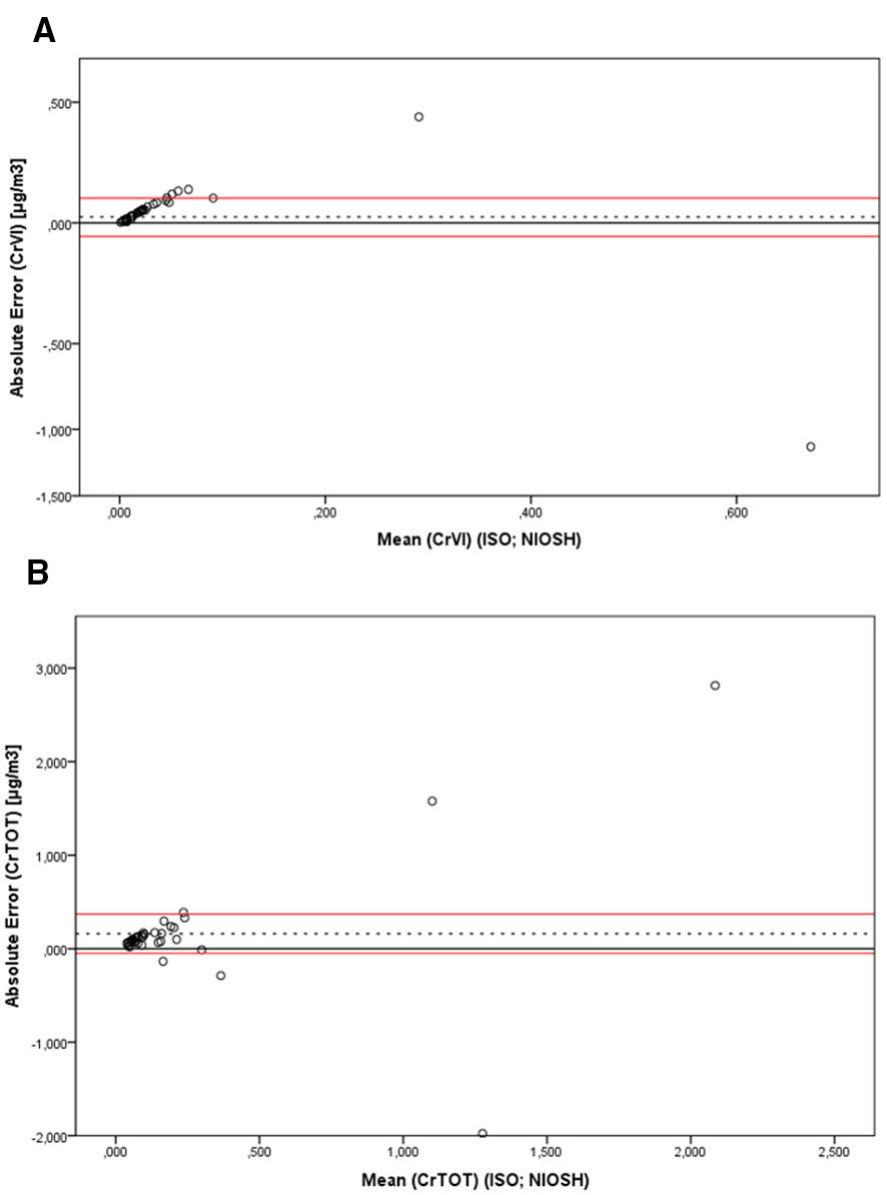
Bland–Altman plots for Cr(VI) **(A)** and CrTOT **(B)**. For each pair of measurements, the absolute error (NIOSH method – ISO method) is plotted against the mean of the two results. The dashed line represents the mean error, while the red lines indicate the 95% confidence limits.

Bland–Altman analysis (Figures 1A–B) showed wide limits of agreement, especially for CrTOT, with variability increasing at higher concentrations. For Cr(VI), most paired measurements clustered near zero difference, with occasional larger deviations.

Compliance with OELs was evaluated following the statistical procedure outlined in EN 689:2019, comparing the UCL of the one-sided 70% confidence interval of the 95th percentile with the corresponding OELs. The applicable 8-h time-weighted average (TWA) limits were 5 μg/m^3^ for Cr(VI) and 500 μg/m^3^ for CrTOT. Most Cr(VI) measurements were well below the OEL, although a few values approached the regulatory threshold. No exceedances were observed for CrTOT.

### Biological monitoring

U-Cr concentrations were significantly higher in exposed workers than in controls both pre-shift (GM 0.34 vs. 0.05 μg/g creatinine; *p* = 0.031; 0.23 vs. 0.04 μg/L) and post-shift (GM 0.36 vs. 0.05 μg/g; *p* = 0.008; 0.27 vs. 0.04 μg/L). In contrast, EBC-Cr showed no differences between groups at either time point (GM 0.08 μg/L; *p* > 0.90) (Table 5). Among exposed workers, neither biomarker changed significantly from pre- to post-shift (U-Cr *p* = 0.874; EBC-Cr *p* = 0.867). Age, residence, years of employment, shift type, welding technique, base material, and LEV were not associated with biomarker variability (Table 6).

**Table 5:** Comparison of urinary chromium (U-Cr) and exhaled breath condensate chromium (EBC-Cr) concentrations between exposed workers and controls during pre-shift and post-shift sampling.

Biomarker	Sampling time	***N***		GM (95% CI)		*p*-value
		Exposed	Control	Exposed	Control	
U-Cr	pre-shift	22	10	0.34 (0.13, 0.85)	0.05 (0.006, 0.41)	**0.031**
	post-shift	22	10	0.36 (0.20, 0.63)	0.05 (0.006, 0.41)	**0.008**
EBC-Cr	pre-shift	3	10	0.08 (0.01, 0.43)	0.08 (0.03, 0.21)	0.937
	post-shift	21	10	0.08 (0.06, 0.13)	0.08 (0.03, 0.21)	0.983

Data are reported as number of subjects (*N*), percentage (%), geometric mean (GM), and 95% confidence interval (95% CI). U-Cr concentrations are expressed as μg/g creatinine (single sample for controls; beginning of the workweek and end-of-shift at the end of the workweek for exposed workers). EBC-Cr concentrations are expressed as μg/L (single sample for controls; end-of-shift at the end of the workweek for exposed workers, with an additional subset of spot samples collected during the workweek). Statistically significant differences between workers and controls (*p* < 0.05) are indicated in bold.

**Table 6:** Evaluation of urinary chromium (U-Cr) and exhaled breath condensate chromium (EBC-Cr) concentrations across demographic and occupational variables, as well as technical and process-related welding characteristics.

Variable	Category	Biomarker/sampling time	*n* (%)	GM (95% CI)	Median (IQR)	P95	*p-value*
Welding activity	Pre-shift	U-Cr	22 (100)	0.34 (0.13, 0.86)	0.49 (0.16, 1.12)	7.42	0.874
	Post-shift		22 (100)	0.36 (0.21, 0.64)	0.39 (0.25, 0.83)	1.87	
	Pre-shift	EBC-Cr	3 (100)	0.08 (0.01, 0.44)	0.08 (0.04, 0.16)	0.16	0.867
	Post-shift		21 (100)	0.08 (0.06, 0.13)	0.11 (0.05, 0.16)	0.38	
Age	< 40	U-Cr pre-shift	13 (59)	0.18 (0.05, 0.73)	0.31 (0.14, 0.84)	1.88	0.144
	≥40		9 (41)	0.84 (0.29, 2.41)	0.74 (0.33, 3.22)	7.84	
	< 40	U-Cr post-shift	13 (59)	0.26 (0.11, 0.65)	0.36 (0.23, 0.66)	1.68	0.110
	≥40		9 (41)	0.59 (0.36, 0.96)	0.49 (0.34, 0.92)	1.90	
	< 40	EBC-Cr post-shift	13 (62)	0.10 (0.05, 0.18)	0.12 (0.06, 0.18)	0.39	0.210
	≥40		8 (38)	0.07 (0.04, 0.14)	0.06 (0.04, 0.12)	0.28	
Residence	Urban	U-Cr pre-shift	13 (59)	0.41 (0.10, 1.75)	0.54 (0.16, 1.63)	7.84	0.431
	Rural area		9 (41)	0.25 (0.07, 0.91)	0.31 (0.17, 0.89)	1.47	
	Urban	U-Cr post-shift	13 (59)	0.37 (0.34, 1.04)	0.49 (0.28, 0.92)	1.90	0.163
	Rural area		9 (41)	0.36 (0.25, 0.54)	0.36 (0.24, 0.48)	0.83	
	Urban	EBC-Cr post-shift	12 (57)	0.08 (0.04, 0.17)	0.12 (0.04, 0.19)	0.39	0.421
	Rural area		9 (43)	0.09 (0.06, 0.13)	0.11 (0.05, 0.14)	0.18	
Smoke	Smokers	U-Cr pre-shift	16 (73)	0.28 (0.08, 1.01)	0.43 (0.14, 1.00)	7.84	0.641
	No smokers		6 (27)	0.55 (0.20, 1.46)	0.49 (0.24, 1.50)	1.88	
	Smokers	U-Cr post-shift	16 (73)	0.28 (0.14, 0.58)	0.35 (0.24, 0.74)	1.00	**0.029**
	No smokers		6 (27)	0.70 (0.30, 1.67)	0.66 (0.34, 1.73)	1.90	
	Smokers	EBC-Cr post-shift	16 (76)	0.07 (0.04, 0.10)	0.07 (0.04, 0.12)	0.19	**0.002**
	No smokers		5 (24)	0.20 (0.11, 0.37)	0.18 (0.13, 0.34)	0.39	
Years of employment	< 15	U-Cr pre-shift	15 (68)	0.22 (0.06, 0.82)	0.31 (0.13, 1.04)	7.84	0.142
	>15		7 (32)	0.83 (0.33, 2.07)	0.74 (0.53, 1.37)	5.08	
	< 15	U-Cr post-shift	15 (68)	0.29 (0.13, 0.64)	0.36 (0.24, 0.82)	1.68	0.210
	>15		7 (32)	0.59 (0.31, 1.12)	0.41 (0.33, 1.00)	1.90	
	< 15	EBC-Cr post-shift	15 (71)	0.09 (0.05, 0.14)	0.12 (0.04, 0.15)	0.39	0.850
	>15		6 (29)	0.08 (0.03, 0.23)	0.09 (0.05, 0.20)	0.28	
Types of work shifts	Fixed Day shift	U-Cr pre-shift	6 (27)	0.24 (0.01, 7.73)	0.55 (0.08, 2.99)	7.84	0.957
	Day/Evening shift		6 (27)	0.56 (0.14, 2.34)	0.58 (0.16, 2.05)	5.08	
	Day/Evening/Night shift		10 (45)	0.31 (0.09, 1.02)	0.43 (0.19, 0.93)	1.88	
	Fixed Day shift	U-Cr post-shift	6 (27)	0.20 (0.02, 2.07)	0.42 (0.04, 1.10)	1.90	0.872
	Day/Evening shift		6 (27)	0.42 (0.22, 0.80)	0.34 (0.25, 0.86)	1.00	
	Day/Evening/Night shift		10 (45)	0.48 (0.31, 0.76)	0.43 (0.31, 0.83)	1.68	
	Fixed Day shift	EBC-Cr post-shift	5 (24)	0.05 (0.01, 0.20)	0.07 (0.02, 0.12)	0.12	0.346
	Day/Evening shift		6 (29)	0.09 (0.04, 0.22)	0.12 (0.05, 0.19)	0.19	
	Day/Evening/Night shift		10 (48)	0.11 (0.06, 0.19)	0.12 (0.05, 0.20)	0.39	
Localized extraction systems	Yes	U-Cr pre-shift	15 (68)	0.31 (0.08, 1.22)	0.53 (0.16, 1.37)	7.84	0.837
	No		7 (32)	0.40 (0.16, 1.04)	0.45 (0.16, 1.04)	1.88	
	Yes	U-Cr post-shift	15 (68)	0.32 (0.14, 0.73)	0.41 (0.24, 0.83)	1.90	0.945
	No		7 (32)	0.47 (0.24, 0.90)	0.34 (0.26, 0.84)	1.68	
	Yes	EBC-Cr post-shift	14 (67)	0.10 (0.04, 0.12)	0.10 (0.04, 0.15)	0.19	0.150
	No		7 (33)	0.12 (0.05, 0.26)	0.12 (0.06, 0.28)	0.39	
RPE	Yes	U-Cr pre-shift	12 (55)	0.25 (0.05, 1.42)	0.43 (0.14, 1.00)	7.84	0.821
	No		10 (45)	0.48 (0.23, 0.98)	0.49 (0.19, 1.40)	1.88	
	Yes	U-Cr post-shift	12 (55)	0.23 (0.09, 0.57)	0.28 (0.22, 0.50)	1.00	**0.036**
	No		10 (45)	0.65 (0.41, 1.04)	0.65 (0.36, 1.05)	1.90	
	Yes	EBC-Cr post-shift	12 (57)	0.07 (0.04, 0.12)	0.09 (0.04, 0.13)	0.17	0.093
	No		9 (43)	0.12 (0.06, 0.23)	0.12 (0.05, 0.23)	0.39	
Base material	Stainless steel	U-Cr pre-shift	17 (77)	0.42 (0.14, 1.23)	0.53 (0.19, 1.42)	7.84	0.244
	Carbon steel		4 (18)	0.10 (0.003, 2.93)	0.23 (0.04, 0.48)	0.52	
	Titanium		1 (5)	1.04 (/)	1.04 (1.04, 1.04)	1.04	
	Stainless steel	U-Cr post-shift	17 (77)	0.37 (0.18, 0.77)	0.37 (0.28, 0.83)	1.90	0.640
	Carbon steel		4 (18)	0.38 (0.20, 0.70)	0.43 (0.26, 0.50)	0.51	
	Titanium		1 (5)	0.25 (,)	0.25 (0.25, 0.25)	0.25	
	Stainless steel	EBC-Cr post-shift	16 (76)	0.08 (0.05, 0.14)	0.12 (0.05, 0.17)	0.39	0.995
	Carbon steel		4 (19)	0.09 (0.03, 0.25)	0.10 (0.05, 0.16)	0.17	
	Titanium		1 (5)	0.11 (/)	0.11 (0.11, 0.11)	0.11	
Welding type	Manual TIG	U-Cr pre-shift	11 (50)	0.35 (0.07, 1.73)	0.53 (0.21, 1.47)	5.08	0.286
	Automatic TIG		3 (14)	0.31 (0.02, 5.18)	0.26 (0.11, 1.04)	1.04	
	MMA		4 (18)	1.11 (0.09, 14.34)	1.14 (0.34, 6.22)	7.84	
	ESW		4 (18)	0.10 (0.003, 2.94)	0.23 (0.04, 0.48)	0.53	
	Manual TIG	U-Cr post-shift	11 (50)	0.25 (0.09, 0.76)	0.33 (0.24, 0.83)	1.68	0.077
	Automatic TIG		3 (14)	0.31 (0.19, 0.53)	0.34 (0.25, 0.36)	0.36	
	MMA		4 (18)	1.07 (0.57, 2.00)	0.92 (0.82, 1.68)	1.90	
	ESW		4 (18)	0.38 (0.20, 0.70)	0.43 (0.26, 0.50)	0.51	
	Manual TIG	EBC-Cr post-shift	11 (52)	0.09 (0.04, 0.18)	0.12 (0.06, 0.15)	0.39	0.973
	Automatic TIG		3 (14)	0.08 (0.01, 0.79)	0.11 (0.03, 0.18)	0.18	
	MMA		3 (14)	0.06 (0.003, 1.07)	0.07 (0.02, 0.19)	0.19	
	ESW		4 (19)	0.09 (0.03, 0.25)	0.10 (0.05, 0.16)	0.17	
Similar Exposure Groups	SEG A	U-Cr pre-shift	11 (50)	0.38 (0.07, 2.13)	0.65 (0.16, 1.37)	7.83	0.652
	SEG B		11 (50)	0.31 (0.11, 0.88)	0.33 (0.21, 0.74)	1.89	
	SEG A	U-Cr post-shift	11 (50)	0.28 (0.09, 0.88)	0.34 (0.25, 0.83)	1.90	0.699
	SEG B		11 (50)	0.47 (0.31, 0.70)	0.41 (0.33, 0.83)	1.68	
	SEG A	EBC-Cr post-shift	10 (48)	0.06 (0.03, 0.12)	0.09 (0.03, 0.12)	0.19	0.118
	SEG B		11 (52)	0.12 (0.07, 0.19)	0.12 (0.06, 0.18)	0.39	

Data are reported as number of subjects (*N*), percentage (%), geometric mean (GM), 95% confidence interval (95% CI), median, interquartile range (IQR), and 95th percentile (P95). U-Cr concentrations are expressed as μg/g creatinine (beginning of the workweek and end-of-shift at the end of the workweek for exposed workers), while EBC-Cr concentrations are expressed as μg/L (end-of-shift at the end of the workweek for exposed workers). Significant differences between workers and controls (*p* < 0.05) are indicated in bold.

Smoking was associated with lower post-shift Cr levels: U-Cr GM 0.28 vs. 0.70 μg/g creatinine (*p* = 0.029; 0.20 vs. 0.56 μg/L) and EBC-Cr 0.07 vs. 0.20 μg/L (*p* = 0.002). Respirators use also reduced post-shift U-Cr (GM 0.23 vs. 0.65 μg/g; *p* = 0.036; 0.17 vs. 0.47 μg/L), while differences in EBC-Cr did not reach significance (*p* = 0.093). SEG-based comparisons showed no significant differences between companies.

Overall, Cr biomarkers were broadly similar across demographic and occupational subgroups, with U Cr consistently distinguishing exposed workers from controls, while EBC Cr showed limited discriminatory capacity (Table 6).

Spearman correlations (Table 7) indicated a moderate, non-significant association between airborne CrTOT and post-shift U-Cr (ISO rs = 0.41; *p* = 0.060; NIOSH rs = 0.38; *p* = 0.086). Correlations with EBC-Cr were weak, both for CrTOT (ISO rs = 0.07; NIOSH rs = 0.28) and between U-Cr and EBC-Cr (rs = 0.29). Filler-material Cr content showed weak or moderate, non-significant correlations with biomarkers.

**Table 7:** Spearman correlation analyses between chromium biomarkers and exposure-related variables.

Comparison	*N*	r_s_	95% CI	*p*-value
CrTOT (ISO) vs. U-Cr post-shift	22	0.41	−0.03, 0.71	0.060
CrTOT (NIOSH) vs. U-Cr post-shift	21	0.38	−0.07, 0.70	0.086
CrTOT (ISO) vs. EBC-Cr post-shift	21	0.07	−0.38, 0.50	0.748
CrTOT (NIOSH) vs. EBC-Cr post-shift	20	0.28	−0.20, 0.65	0.229
U-Cr post-shift vs. EBC-Cr post-shift	21	0.29	−0.18, 0.65	0.205
% Cr (filler material) vs. U-Cr post-shift	16	−0.23	−0.66, 0.31	0.389
% Cr (filler material) vs. EBC-Cr post-shift	15	0.36	−0.20, 0.74	0.184
% Cr (filler material) vs. air-Cr (ISO)	16	0.13	−0.40, 0.60	0.638
% Cr (filler material) vs. air-Cr (NIOSH)	15	−0.07	−0.57, 0.47	0.798

Data are reported as sample size (*N*), Spearman's rank correlation coefficient (r_s_), and 95% confidence interval (95% CI) for urinary chromium concentrations (μg/g creatinine; single sample for controls; beginning of the workweek and end-of-shift at the end of the workweek for exposed workers) and for chromium concentrations in exhaled breath condensate (EBC, μg/L; single sample for controls; end-of-shift at the end of the workweek for exposed workers, with an additional subset of spot samples collected during the week).

**Figure 2:**
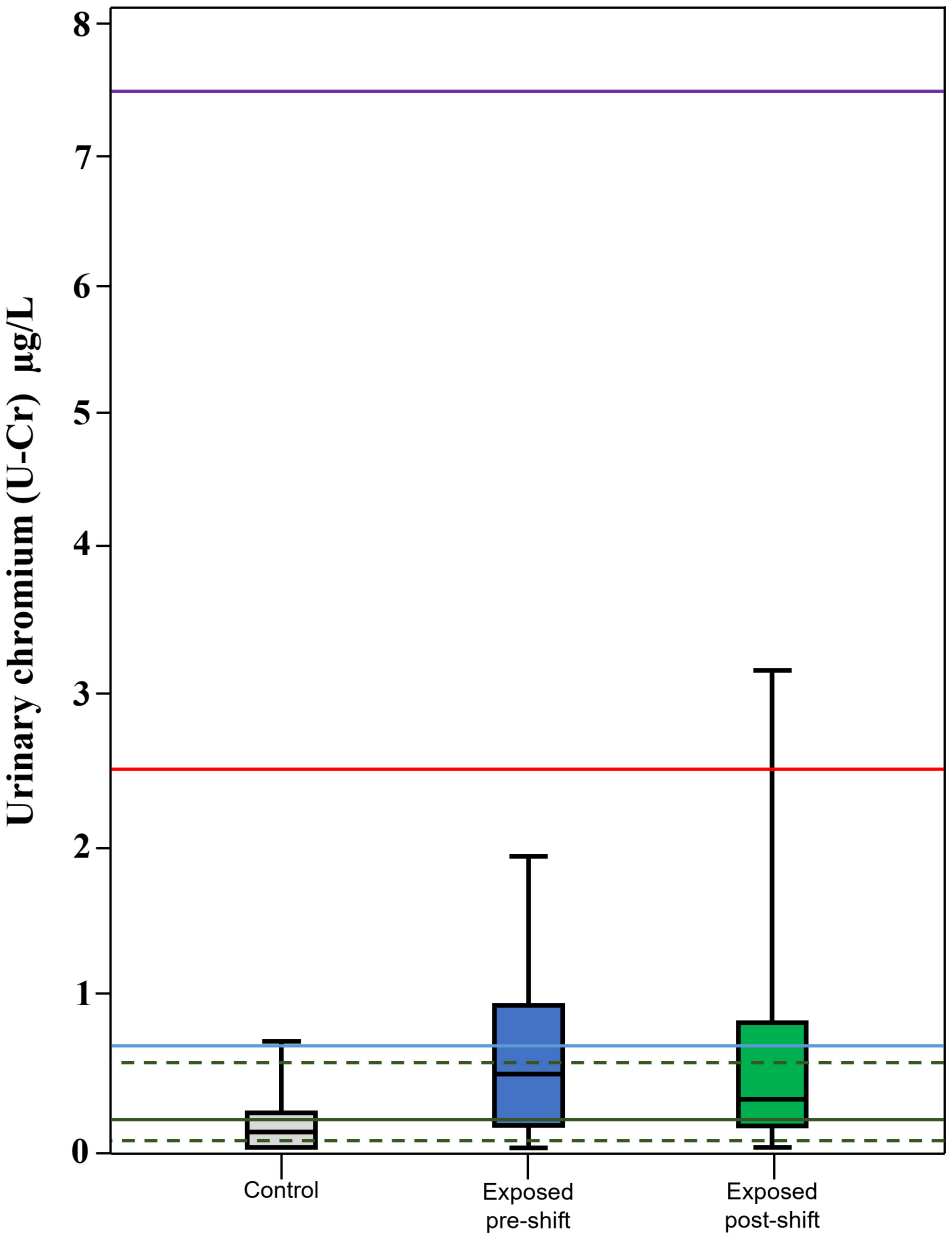
Boxplots of U–Cr (μg/L) for controls and exposed workers, separately for pre- and post-sampling measurements. Horizontal green lines indicate the reference values for the general adult population proposed by SIVR: the solid green line corresponds to the geometric mean (0.22 μg/L), and the dashed green lines represent the 5th and 95th percentiles (0.05 and 0.60 μg/L, respectively) and DFG BAR (0.60 μg/L). The horizontal light blue line represents the ACGIH BEI (0.70 μg/L). The horizontal red line represents the occupational BLV proposed by ANSES for end-of-shift, end-of-week (2.50 μg/L). The horizontal purple line represents the BLV proposed by INSST (7.50 μg/L).

Compared with population reference values (SIVR GM 0.22 μg/L; 5th−95th percentile 0.05–0.60 μg/L), controls aligned with expected background levels (GM 0.04 μg/L), whereas welders showed elevated P95 values both pre-shift (1.96 μg/L) and post-shift (2.94 μg/L) (Figure 2).

Overall, the data indicate that welders experience Cr uptake above general-population background, though still within a low-exposure scenario relative to international limits.

## Discussion

Occupational exposure to welding fumes, particularly those containing Cr(VI), remains a key concern in industrial hygiene. Despite increasingly stringent regulatory limits (16, 17), real-world assessments still report relevant exposures, especially during stainless-steel welding or in settings with inadequate ventilation (18). Integrated environmental and biological monitoring approaches, promoted by initiatives such as HBM4EU (10, 11, 19, 20), support more harmonized and informative exposure assessment. Within this framework, the present study combines environmental and biological monitoring to characterize chromium exposure among welders and to evaluate how analytical methods and process-specific factors influence exposure classification.

### Environmental exposure and determinants

From an environmental perspective, airborne Cr concentrations were consistently higher among welders than controls, confirming occupationally relevant exposures even where engineering controls were generally adequate. MMA welding showed higher Cr(VI) levels than TIG, in line with established emission profiles: MMA produces more fumes due to consumable coated electrodes, whereas TIG generates lower particulate emissions (21). Although most Cr(VI) values remained below the 8-h TWA OEL of 5 μg/m^3^, some measurements approached this limit, indicating episodic peaks and reinforcing the need for continuous exposure minimization for Group 1 carcinogens such as Cr(VI), for which no safe threshold can be assumed. A key contribution of this study is the comparison between the NIOSH 7600 protocol and a modified ISO 17075 method optimized for Cr speciation. Analytical methodology strongly influenced measured Cr(VI) levels and exposure classification. As a result, the higher Cr(VI) values obtained with NIOSH 7600 should be interpreted cautiously. They are consistent with a potential positive bias arising from partial oxidation of Cr(III) to Cr(VI) during the alkaline extraction step-a known limitation of this method. NIOSH 7600, in fact, based on hot alkaline extraction, may overestimate Cr(VI) due to partial solubilization and oxidation of Cr(III) and metallic Cr, elevating background levels and reducing discrimination between exposed workers, controls, and welding techniques. However, the present study did not directly assess species interconversion, and no mechanistic inference can be drawn from these data. For CrTOT, variability was more evident: at lower concentrations the two methods produced closely aligned results, whereas discrepancies became progressively larger as concentrations increased. This pattern points to a proportional component in the inter-method differences, suggesting that disagreement between methods grows with the amount of analyte collected, as expected for measurements whose analytical uncertainty scales with concentration. The modified ISO 17075 protocol, operating under near-neutral pH and ambient temperature, reduces oxidation artifacts and provides clearer differentiation between exposure groups. Using this method, welders - especially those performing MMA - showed higher Cr(VI) levels than controls, consistent with expected emission patterns (21). Bland–Altman analysis indicated wide limits of agreement between methods, particularly for total Cr, with increasing dispersion at higher concentrations. For Cr(VI), discrepancies were generally within a few hundred ng/m^3^ but occasionally larger. These results suggest that part of the between-worker variability may stem from methodological differences rather than true differences in inhaled dose. Consequently, reliance on NIOSH 7600 may introduce non-differential misclassification, attenuating contrasts between welding techniques. In contrast, the modified ISO 17075 method improves exposure classification within the concentration range relevant to this cohort, offering more specific Cr(VI) quantification and a more robust basis for risk assessment. Adoption of speciation-preserving analytical approaches can therefore enhance exposure characterization and support more targeted preventive strategies.

### Biological monitoring and biomarker performance

Biological monitoring confirmed occupational uptake of chromium. Urinary chromium (U-Cr) levels were significantly higher in welders than controls, both pre- and post-shift, whereas EBC-Cr showed no group differences. The interpretation of the biological monitoring data requires careful consideration. For U-Cr, the absence of a clear pre/post-shift increase may indicate that urinary chromium reflects exposure integrated over several days, thereby smoothing short-term fluctuations. However, this pattern could also be influenced by limited within-shift variability in airborne Cr exposure among welders and by the modest statistical power associated with the sample size (22). This interpretation is partly supported by the weak-to-moderate correlations observed between airborne CrTOT (ISO and NIOSH) and post-shift U-Cr (rs = 0.41 and 0.38), with *p*-values approaching significance, suggesting a biologically plausible link between inhaled Cr and urinary excretion. Nonetheless, these associations did not reach conventional statistical significance and should therefore be regarded as suggestive rather than conclusive. The interpretation of EBC-Cr presents additional challenges. Although EBC has theoretical value as a non-invasive matrix that could reflect Cr species in the respiratory tract, this biomarker showed poor ability to distinguish between exposed and control subjects. This likely reflects a combination of biological and methodological constraints. From a biological perspective, the fraction of Cr that partitions into the airway lining fluid may be small and weakly correlated with inhaled dose. EBC, in fact, reflects a local and dynamic airway compartment and may therefore be influenced by recent particle deposition, local redox transformations, and short-term clearance processes, rather than cumulative systemic uptake alone (13, 23). Methodologically, the relatively high LOQs, the analytical variability inherent to trace-level measurements, and the absence of standardized procedures for dilution correction in EBC all contribute to uncertainty and may hinder true exposure-related differences. Taken together, these factors may explain the limited discriminatory performance of EBC-Cr in this study and highlight that both biological and analytical constraints play a role. Overall, these results suggest U-Cr is a more robust biomarker of systemic uptake, whereas EBC is less sensitive at low-to-moderate exposure levels (23).

The HBM4EU chromates study (10) and Leese et al. (11) reported very low EBC- Cr levels, with most values below the LOQ and P95 values for Cr(VI) ranging from 0.14 to 0.74 μg/L and Cr(III) medians of 0.12–0.17 μg/L (P95: 0.35–0.46 μg/L). Among Italian welders in HBM4EU, EBC-Cr(VI) was almost always below the LOQ (0.05 μg/L), with maxima around 0.33 μg/L. Our findings align with this pattern, confirming that EBC-based biomarkers remain unsuitable for routine monitoring, whereas U-Cr is more reliable. In HBM4EU welders, post-shift U-Cr medians reached 0.68 μg/g creatinine (P95: 3.36 μg/g), while our cohort showed lower central tendency and upper percentiles, placing them at the lower end of contemporary European exposures.

When demographic and lifestyle variables were considered, age stratification showed higher U-Cr geometric means among older welders (≥40 years), a pattern consistent with longer cumulative exposure and, potentially, with more frequent assignment to higher-emission tasks. Age-related differences in the control group were minimal. These trends are broadly in line with HBM4EU findings, where welders exhibited U-Cr distributions shifted above controls and associated with airborne Cr(VI) levels. Regarding smoking status, smokers showed lower post-shift U-Cr and EBC-Cr levels than non-smokers. This unexpected pattern is unlikely to reflect a biological effect of smoking and more plausibly arises from residual confounding - such as differences in work practices or task allocation - inter-individual variability, or chance given the limited sample size. Because of the small cohort, no formal multivariable modeling was feasible, as fully adjusted models would have been unstable and at risk of overfitting. Future studies with larger samples and more detailed task-based exposure characterisation will be essential to disentangle lifestyle influences from occupational determinants and to clarify the true contribution of demographic factors to biomarker variability.

The Cr content of filler materials showed only weak, non-significant associations with airborne or biological metrics, likely reflecting the multifactorial determinants of fume composition-including welding parameters, ventilation efficiency, and metal transfer mechanisms (24). When benchmarked against population reference values, U-Cr clearly indicated occupational contribution. Controls largely fell within background ranges, whereas welders exceeded the SIVR 95th percentile (0.60 μg/L) (25), with P95 values of 1.96 μg/L (pre-shift) and 2.94 μg/L (post-shift). Welders' U-Cr distributions were shifted above population reference ranges and above controls. However, group geometric means (0.23 and 0.27 μg/L) remained below occupational biological limits, including the ACGIH BEI (0.7 μg/L) (26), DFG BAR (0.6 μg/L) (27), BLVs proposed by ANSES (2.5 μg/L) (28) and INSST (7.5 μg/L) (29). Overall, welders showed elevated but low-range occupational exposure, with biomarker increases driven primarily by workplace factors, consistent with age-related trends and the absence of smoking effects.

### Strengths, limitations, and implications for occupational health surveillance

While this study provides a coherent picture of Cr exposure in welding settings, several limitations must be acknowledged. A major limitation of this study is the relatively small sample size, which, although common in occupational field research, inevitably reduces statistical power. This constraint is particularly relevant for subgroup comparisons and correlation analyses, where modest associations may remain undetected. As a result, non-significant or borderline findings should be interpreted with caution, as they may reflect insufficient power rather than the absence of a true relationship. The exploratory nature of the study and the limited number of participants therefore restrict the generalizability of the results, which should be confirmed in larger and more adequately powered cohorts. In addition, the cross-sectional design of the study limits the ability to evaluate temporal dynamics, intra-individual variability, or causal relationships between exposure and biomarker responses. Without repeated measures or longitudinal follow-up, it is not possible to assess how fluctuations in exposure influence biomarker kinetics over time. Future studies adopting longitudinal or mixed-design approaches would allow a more refined characterisation of exposure–response relationships and strengthen causal inference.

Because the sample size was not adequate to support stable adjusted analyses, no multivariable models were applied. Fully adjusted models would likely have been unstable and at risk of overfitting; therefore, all associations must be regarded as hypothesis-generating rather than conclusive. In addition, participation was voluntary, and some degree of selection bias cannot be excluded. Residual confounding from non-occupational chromium sources, lifestyle factors, or inter-individual variability may also persist. For example, the lower U-Cr levels observed among smokers should not be overinterpreted, as this unexpected pattern may reflect differences in work practices, task allocation, or random variation rather than a true biological effect.

The biomonitoring panel was also limited. Recent HBM4EU evidence highlights the added value of multiple complementary biomarkers, including chromium in red blood cells (RBC-Cr), which provides more specific information on intracellular Cr(VI) bioavailability beyond urinary excretion, as well as biomarkers of oxidative stress and inflammation that capture early biological effects. Their absence reduces the ability to fully characterize exposure pathways and biological responses. The observed correlations between airborne Cr and U-Cr were biologically plausible but weak to moderate and did not reach statistical significance; they should therefore be considered suggestive rather than conclusive. Given the limited sample size and the cross-sectional design, these associations may reflect random variability, insufficient statistical power, or unmeasured confounding rather than a true exposure–response relationship. While the direction of the correlations is consistent with expected toxicokinetic pathways, the magnitude and precision of the estimates do not support firm inferences. Accordingly, these findings warrant further investigation in larger, longitudinal studies with more comprehensive exposure characterisation and expanded biomarker panels.

Methodological constraints further affect the interpretation of EBC-based indicators. Chromium concentrations in EBC were frequently low and close to the LOQ, a finding consistent with multicentre investigations and indicative of the analytical challenges inherent to this matrix. Moreover, the absence of a validated dilution marker prevents correction for inter-sample variability in respiratory water content, while the lack of routine Cr(III)/Cr(VI) speciation limits the capacity to distinguish between Cr species with differing toxicological relevance. These methodological constraints, combined with the limited fraction of inhaled Cr expected to partition into the airway lining fluid, substantially reduce the sensitivity and interpretability of EBC-derived measurements. In this context, and in line with recent HBM4EU evidence, EBC-Cr should be regarded as an exploratory indicator rather than a validated biomarker for occupational surveillance. Further methodological advancements - such as improved analytical sensitivity, standardized dilution-correction strategies, and speciation-resolved analyses - will be essential to clarify the potential role of EBC in chromium biomonitoring.

Taken together, these limitations indicate that the present findings should be viewed as preliminary. Future research would benefit from larger, longitudinal cohorts, more detailed task-based exposure characterisation, and expanded biomarker panels - including RBC-Cr and effect biomarkers - alongside improved methodological standardization for EBC collection and analysis. Such developments will be essential to strengthen causal inference and refine the role of biological monitoring in assessing chromium exposure among welders.

No multivariable models were applied because the sample size was not adequate to support stable adjusted analyses. Accordingly, non-significant and borderline findings should be interpreted cautiously and regarded as hypothesis-generating rather than conclusive. In addition, because participation was voluntary, some degree of selection bias cannot be excluded, and residual confounding from non-occupational Cr sources and inter-individual variability cannot be ruled out. Similarly, the lower U-Cr levels observed among smokers should not be overinterpreted and may reflect residual confounding, differences in work practices, or chance. The biomonitoring panel was also limited. Recent HBM4EU evidence highlights the added value of multiple biomarkers, including Cr in red blood cells (RBC-Cr), which provides more specific information on intracellular Cr(VI) bioavailability beyond urinary excretion, as well as biomarkers of oxidative stress and inflammation (14, 30). Methodological constraints also affect EBC: Cr concentrations were frequently low and near the LOQ, consistent with multicenter findings (10, 11). The absence of a dilution marker and routine Cr(III)/Cr(VI) speciation further complicates interpretation (11), indicating that EBC-based indicators should be considered exploratory tools rather than validated biomarkers for routine surveillance.

Despite these limitations, the study has important strengths. Environmental monitoring followed EN 689:2019 (15), ensuring robust assessment of OEL compliance. The dual analytical approach-comparing NIOSH 7600 with a modified ISO 17075 method-enabled explicit evaluation of methodological influences on exposure quantification, demonstrating that analytical choice can materially affect classification, especially at low concentrations relevant for regulatory decisions. Integrated monitoring also identified individual deviations, including some controls exceeding background reference values and welders approaching proposed BLVs, illustrating the practical utility of combined environmental and biological data for real-world surveillance. Nevertheless, it is worth noting that the integrated environmental and biological monitoring proved essential for identifying exposure patterns that would not emerge from either approach alone. Environmental measurements capture airborne concentrations and process-related determinants, while biomonitoring reflects internal dose and incorporates individual factors such as work practices, PPE use, ventilation, and physiological variability - crucial for Group 1 carcinogens like Cr(VI). In this study, the combined interpretation of airborne Cr (measured with speciation-preserving methods) and EBC-Cr and U-Cr levels benchmarked against reference values enabled a more accurate exposure classification and clearer distinction between occupational and background contributions. This integrated framework strengthens evaluation of control-measure effectiveness and supports timely risk characterization and preventive actions.

## Conclusions

This study characterized occupational exposure to Cr in welders, with a focus on accurate Cr(VI) determination through environmental monitoring. The combined use of conventional and advanced analytical protocols demonstrated the feasibility and added value of integrating refined exposure-assessment methods into occupational risk evaluation. This study should be interpreted as an exploratory cross-sectional investigation and does not allow causal inference or assessment of longer-term exposure trajectories. Despite its exploratory nature, the study provides meaningful insight into real-world Cr exposure patterns and highlights the importance of analytical approaches capable of distinguishing Cr species to improve risk characterization and monitoring strategies in Cr(VI)-relevant industries. Integrated environmental and biological monitoring, conducted within a rigorous framework (EN 689 compliance and dual analytical methods), suggested that U-Cr was the most informative biomarker of systemic chromium uptake within the limits of this study, consistently exceeding population background levels while remaining below proposed BLVs. In contrast, EBC-Cr showed limited suitability for routine surveillance, reinforcing the need for continued exposure minimization according to the ALARA principle for this Group 1 carcinogen. Future research should broaden the biomarker panel to include additional exposure and early-effect indicators and improve EBC methodology through standardized dilution markers, routine speciation, and enhanced analytical sensitivity. Larger multicenter and longitudinal studies integrating process-specific environmental monitoring with harmonized biomonitoring frameworks are needed to clarify exposure determinants and cumulative patterns, particularly in low-to-moderate exposure scenarios. These developments will strengthen the scientific basis for occupational risk assessment and prevention in welding and other Cr(VI) -exposed sectors.

## Data Availability

The original contributions presented in this study are included in the article and supplementary material. Further inquiries can be directed to the corresponding author.
